# Tobacco and Clear Thinking

**Published:** 1919-01-18

**Authors:** 


### TOBACCO AND CLEAR THINKING.

The following letter appears in the *British Medical Journal* of December 28:—

Tobacco and Neurasthenia.

"M. B." writes: Since the commencement of the war tobacco has obtained far too great a hold upon the community generally, but I doubt whether the medical profession has fully appreciated the craving which neurasthenics have for tobacco, and especially in the form of cigarettes—a most prejudicial vicious circle becomes established, and, as one patient so truly confided to me, the inhalation of cigarettes is one of the causes of this disability, and neurotic patients who are candid with themselves and their medical advisers recognise this fact; but unfortunately the loss of self-control prevents the breaking of a habit. I would appeal to the medical profession to assist their neurasthenic patients in overcoming this pernicious state of affairs, which retards improvement, and, I am convinced, prevents a permanent cure in a large precentage of cases.

As an example of unwarranted assumption and of muddleheadedness this effusion is hard to beat. What evidence is there that since the commencement of the war tobacco has obtained far too great a hold upon the community, or any greater hold than it had before? The assertion is a pure assumption. If tobacco has obtained far too great a hold upon anything but the imagination of M.B., there should be some evidence of it. Is there any evidence? If so, what is it? An unsupported assertion is evidence of the state of mind of the asserter, but it is not evidence of anything else.

Have neurasthenics, as such, any greater craving for tobacco than other people? What is the evidence? We know of none. What is the most prejudicial vicious circle, and what is the difference between a prejudicial circle and a vicious circle? Is there any?

Was the confession of the patient true? Did he inhale cigarettes? Who operated on him to extract them from his trachea and bronchi? And how many were extracted? What is the difficulty that is caused by the inhalation of cigarettes? Presumably it is an inability to breathe with comfort, and does it need any remarkable candour to recognise this fact? or what is the fact that patients who are candid with themselves and their medical advisers recognise? Is self-control lost in neurasthenia? Have neurasthenics no self-control at all? What is the evidence? If it is true, it is a wholly new observation; but is it true?

What is the pernicious state of affairs that retards improvements and prevents a permanent cure? Is it the too great a hold that tobacco has obtained upon the community, or is it the prejudicial vicious circle of inhaling cigarettes and presumably having them extracted by operation?

M. B. is convinced that this state of affairs, whether the hold of tobacco, or the habit of inhaling cigarettes, or the confession of the patient, or the prejudicial vicious circle, or the fact, whatever it may be, we are not told, but he is convinced that the state of affairs prevents a permanent cure in a large proportion of cases. Good, but what is the worth of a conviction based upon such grounds?—upon no ground at all as far as appears.

Whether the hold of tobacco, the prejudicial vicious circle, etc., do or do not produce neurasthenia, retard its improvement and prevent its permanent cure, we offer no opinion in the absence of any evidence one way or the other; but if, as we may presume with some confidence, M. B. does not allow tobacco to get hold of him, and does not inhale cigarettes, it would appear that there is some evidence that abstinence from tobacco, if it does not produce neurasthenia, or weakness of nerves, does tend to produce psychasthenia, or weakness of mind. Our counsel to M. B. would be that he, or peradventure she, should try a course of cigarette smoking, being careful not to inhale the cigarettes, even if he, or she, inhales the smoke of them, and see whether this practice would not clear up some of his, or her, muddleheadedness, and enable him or her, if not to refrain from hasty and unwarranted conclusions, at least to write intelligibly.

